# Effects of digital technology-based interventions on balance function and non-motor outcomes in Parkinson’s disease: a systematic review and meta-analysis

**DOI:** 10.3389/fneur.2026.1844772

**Published:** 2026-07-29

**Authors:** Bowen Zheng, Wei Wang, Amanda Lee, Weicui Gao, Xiaolei Wang

**Affiliations:** 1 School of Nursing, Shandong First Medical University & Shandong Academy of Medical Sciences, Tai’an, Shandong, China; 2 Department of Infection Management, Shandong Provincial Hospital Affiliated to Shandong First Medical University, Jinan, Shandong, China; 3 Faculty of Health and Education, School of Nursing and Public Health, Manchester Metropolitan University, Manchester, United Kingdom

**Keywords:** balance function, digital technology, meta-analysis, non-motor outcomes, Parkinson’s disease

## Abstract

**Background:**

Various digital technologies have been adopted for rehabilitation training and extended care in patients with Parkinson’s disease (PD), yet their effectiveness remains unclear. This review aims to evaluate the effectiveness of digital technology-based interventions on balance and non-motor outcomes (cognitive function, depression, and quality of life) in individuals with PD.

**Methods:**

We systematically searched six electronic databases (PubMed, EMBASE, Web of Science, CINAHL, Cochrane Library, and PsycINFO) in March 2026. Two reviewers independently screened randomized controlled trials (RCTs) meeting the inclusion criteria and assessed methodological quality using the Cochrane RoB Tool. We calculated standardized mean differences (SMDs) and 95% confidence intervals (CIs) to pool effect sizes for studies with consistent outcome measures, and performed subgroup analyses by intervention type, duration, and frequency.

**Results:**

We included 29 RCTs with 1,298 PD patients. Meta-analysis revealed that versus the control group, digital technology-based interventions were associated with favorable improvements in balance function (SMD = 0.62, 95%CI 0.16 to 1.09) and cognitive function (SMD = 0.50, 95% CI 0.18 to 0.83) in patients with PD. No significant improvements were found in depression (SMD = −0.17, 95% CI −0.42 to 0.09) or quality of life (SMD = 0.01, 95% CI −0.15 to 0.16). Exploratory subgroup analyses suggested that virtual reality (SMD = 0.88, *p* = 0.02), robotics (SMD = 0.49, *p* = 0.03), interventions lasting ≥ 8 weeks (SMD = 2.91, *p* < 0.0001), 3 weekly sessions (SMD = 0.36, *p* = 0.03), > 3 weekly sessions (SMD = 2.77, *p* = 0.002), and active exercise training (SMD = 0.75, *p* = 0.006) might be associated with improvements in balance function in PD patients.

**Conclusion:**

Digital technology-based interventions have the potential to enhance balance and cognitive function in PD patients, while their effects on depression and quality of life remain inconclusive. Exploratory subgroup analyses showed that virtual reality, robotic training, intervention duration ≥8 weeks, frequency ≥3 sessions per week, and active exercise training were associated with improvements in balance function, though these findings should be considered hypothesis-generating. Given the high heterogeneity, unstable sensitivity analysis for the primary outcome, high risk of performance bias across all trials, and unclear allocation concealment in most studies, the current evidence remains preliminary, and its clinical value requires confirmation through further high-quality research.

**Systematic review registration:**

https://www.crd.york.ac.uk/PROSPERO/view/CRD42024628620, identifier (CRD42024628620).

## 1 Introduction

The global burden of Parkinson’s disease (PD) is expected to increase in line with the global aging population demographic (1, 2). PD is characterized by two types of symptoms: motor and non-motor. Motor symptoms are characterized by postural instability, bradykinesia, and resting tremor (3). Among these, postural instability becomes increasingly prevalent in mid-to-late-stage patients with PD (4). Non-motor symptoms, on the other hand, include cognitive impairment, sleep disorders, and mental dysfunction (3). Such clinical manifestations not only impair patients’ quality of life but also raise their financial burden and influence their mental health. According to statistics, more than one-third of PD patients are troubled by depressive symptoms (5). For patients with PD, rehabilitation and continuity of care are crucial.

Traditional rehabilitation therapies led by rehabilitation therapists in clinical settings face several challenges, including an insufficient number of therapists, a lack of experienced professionals, long training cycles, and poor patient compliance (6). These challenges are even more pronounced among outpatients. Forty percent of PD patients do not visit a neurologist within a full year, and only 9.1% consult a movement disorder specialist (MDS). Few PD patients receive rehabilitation therapies such as physical therapy and occupational therapy (7).

The WHO’s Global Strategy for Digital Health 2020–2025 clearly emphasizes that digital technology can improve the accessibility, efficiency, and quality of health services (8). Digital technology has become an effective tool to promote healthy behavior change and improve health outcomes, with certain advantages over conventional rehabilitation. Currently, a variety of digital technologies have been applied to rehabilitation exercises and extended care for patients with PD, mainly including virtual reality (9), robotics (10), wearable electronic devices (11), and mobile applications (12).

While digital technologies represented by virtual reality, robotics, wearable electronic devices and mobile applications have been increasingly adopted in the treatment and management of PD, their effectiveness in improving patients’ rehabilitation outcomes remains inconclusive. For example, some randomized controlled trials (RCTs) have shown that digital technology-based interventions are more effective in improving balance (9, 13), quality of life (14), cognitive functioning (15, 16), and depression (17) in patients with PD compared with control interventions. However, other trials have shown that digital technology-based and control groups do not show significant differences in balance (18), cognitive functioning (19), quality of life (11, 20), and depression (12). Existing systematic reviews on digital interventions for PD have several limitations: they focus only on single technologies such as virtual reality and robotics (21, 22), and overlook the potential of other digital techniques. Most evaluate motor symptoms including balance and gait, but neglect non-motor outcomes such as cognitive function and depression (21, 22). Furthermore, the included digital health interventions are broadly classified, with balance only analyzed as part of overall motor symptoms, and no specific exploration of the effects of virtual reality, robotics, wearable electronic devices and mobile applications on balance function (23).

Therefore, this study investigated the impact of digital technology-based interventions, including virtual reality, robotics, wearable electronic devices and mobile applications, on balance function, cognitive function, depression and quality of life among individuals with PD. We performed subgroup analyses according to intervention type, duration, frequency and intervention functional characteristics.

## 2 Methods

This research followed the Equator Network guidance and utilized PRISMA (24) for the systematic review and retrieval processes for meta-analysis of RCTs. The study protocol was submitted and prospectively registered with PROSPERO (Registration No. CRD42024628620).

### 2.1 Inclusion criteria

This systematic review only included RCTs and excluded systematic reviews, meta-analyses, non-randomized controlled trials, and conference abstracts. We included all studies focusing on patients diagnosed with PD and older than 18 years of age, regardless of gender, disease severity, etc. Studies comparing digital interventions (either alone or combined with additional treatments) against any active comparator were considered, including conventional rehabilitation training, treadmill therapy, physiotherapy, pharmacotherapy, and home-based training. We ruled out individuals with other neurological or musculoskeletal disorders or major diseases from the study. When mixed population data were present, disaggregated data were incorporated into the analysis where accessible; the reverse was excluded.

### 2.2 Search strategy and study selection

For this systematic review, we performed a thorough literature search in the following databases up to March 2026: PubMed, EMBASE, Web of Science, CINAHL, Cochrane Library, and PsycINFO. We applied medical subject headings (MeSH) and free-text keywords relevant to this review, and used Boolean operators (AND, OR, NOT) to refine the search outputs. Furthermore, we manually checked the reference lists of selected studies and related reviews for supplementary publications. No language limits were imposed during the search; non-English articles were translated for data extraction. Detailed information about the search strategies and search results of each database is available in Supplementary File 1.

### 2.3 Data extraction

Data extraction from all included studies was performed independently by two reviewers using a standardized and preformatted Excel spreadsheet, and a third reviewer was invited to verify the reliability and consistency of all extracted data. The extracted data covered key study characteristics (author, publication year, country), participant demographic profiles (sample size, mean age), as well as comprehensive details of the intervention and control groups (intervention content, intervention duration, assessment period, etc.). The intervention and control groups were tabulated for comparison. To assert homogeneity of methodology and outcome measures, we extracted data on the outcomes assessed in each study. For continuous outcomes, data were summarized as mean and standard deviation, whereas dichotomous outcomes were expressed as absolute event counts.

### 2.4 Methodological quality appraisal

This study adopted the Cochrane Risk of Bias Tool (RoB 1) for randomized controlled trials (25). This instrument assesses seven key domains associated with bias risk. Each domain was classified as having a low, unclear, or high risk of bias. Any disagreements between the two reviewers were settled through consultation with a third reviewer.

### 2.5 Outcomes

The primary outcome was balance function. Eligible balance assessment tools in this meta-analysis were restricted to comprehensive functional balance measures, including the Berg Balance Scale (BBS), Mini-Balance Evaluation Systems Test (Mini-BESTest), and other relevant validated scales. Functional assessments focusing on single movement and timed performance were excluded, such as the Timed Up and Go (TUG) test, Five-Time Sit-to-Stand Test (FTSTS), and similar timed functional tests. Meanwhile, instruments for evaluating balance control at the neurosensory integration level, such as the Sensory Organization Test (SOT), were also excluded.

Change scores of balance scale measurements (calculated as post-intervention score minus baseline score) were uniformly extracted as the analytical data in this meta-analysis. When a single study employed multiple tools to evaluate balance function, such as BBS and Mini-BESTest, the most frequently used scale across all included studies was selected for pooled analysis to reduce between-study heterogeneity. For scales with inconsistent scoring directions in reflecting balance performance, raw data of the same balance outcome from primary studies were inversely converted referenced to the most commonly applied scale, so as to complete the standardization of outcome metrics.

Secondary outcomes included cognitive function, depression, and quality of life, all of which were non-motor outcomes. Change scores from baseline to post-intervention were extracted for all secondary indicators. The principles of multi-scale selection and scoring direction standardization were consistent with those applied to the primary outcome.

### 2.6 Statistical analysis

We computed standardized mean differences (SMDs) with 95% confidence intervals to compare outcomes across studies using different measurement scales. For studies with identical outcome measures, we calculated mean differences (MDs) and their corresponding 95% confidence intervals. Heterogeneity across studies was evaluated via the I^2^ statistic. When *p* ≥ 0.10 and I^2^ < 50%, no significant heterogeneity was detected, and a fixed-effect model was applied for pooling; in contrast, when *p* < 0.10 and I^2^ ≥ 50%, substantial heterogeneity was observed (26), and a random-effect model was employed instead. All statistical analyses were performed using RevMan 5.4 software, and *p* < 0.05 was considered statistically significant. Publication bias was examined by visually inspecting funnel plots in meta-analyses that included 10 or more trials.

In this systematic review, we conducted subgroup analyses based on intervention type, intervention duration, intervention frequency and intervention functional characteristics to explore heterogeneity and ensure the authenticity and homogeneity of the study findings and methods. In addition, sensitivity analyses were planned to exclude trials judged as having high risk of bias in any domain, or high or unclear risk of bias in the domain of outcome assessor blinding (detection bias).

## 3 Results

### 3.1 Study selection

**Figure 1:**
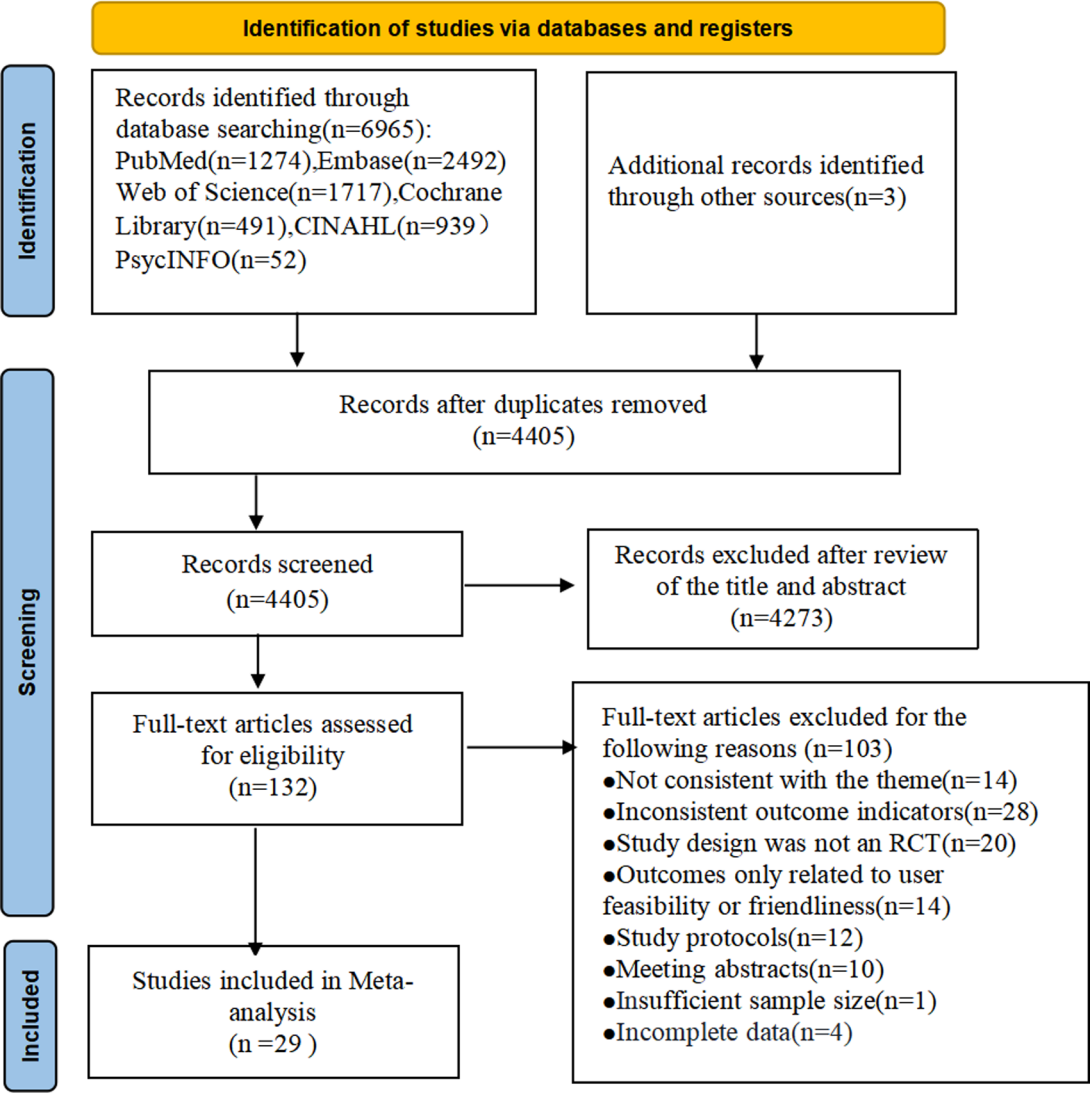
Study selection process following the PRISMA guidelines.

A total of 6,965 records were identified from databases, and an additional 3 records were obtained from other sources. After removing duplicates and irrelevant literature, 132 records remained. Following full-text screening, 103 studies were excluded, leaving 29 eligible studies (9–20, 26–42) involving 1,298 patients with PD. Figure 1 presents the detailed screening process.

### 3.2 Study characteristics

Participant numbers across the studies ranged from 12 to 157, with intervention durations spanning from 4 to 26 weeks. Geographic locations included Italy [13 studies (10, 13, 15, 16, 18, 26, 28, 30, 36–40)], China [3 studies (29, 35, 42)], Turkey [2 studies (14, 32)], Pakistan [2 studies (9, 33)], South Korea [3 studies (17, 20, 34)], the United States [1 study (27)], Belgium [1 study (31)], Brazil [1 study (19)], Portugal [1 study (41)], Spain [1 study (11)], and the United Kingdom [1 study (12)]. All 29 studies reported the inclusion and exclusion criteria for participants. 16 studies applied virtual reality interventions (9, 13, 14, 16, 17, 19, 29, 30, 32, 33, 35–38, 41, 42), 6 studies used robotic training (10, 18, 26, 34, 39, 40), 3 studies adopted wearable electronic devices (11, 27, 28), 1 study used mobile applications (12), 2 studies used a combination of wearable devices and mobile applications (20, 31), and 1 study used a combination of virtual reality and mobile applications (15). All 29 included studies used active control designs. Of these, 18 studies (10, 12–16, 18–20, 27–29, 31, 35, 36, 38, 39, 42) allocated the intervention group to receive digital interventions and the control group to receive conventional training. 7 studies (9, 11, 17, 32, 33, 37, 41) adopted a design where the control group received conventional training alone, and the intervention group received additional digital technology-based interventions on top of conventional training. The remaining 4 studies compared digital interventions with treadmill training (26, 34, 40) or sensory integration balance training (30) in the control group. Detailed characteristics of the included studies are summarized in Supplementary Table S1.

### 3.3 Risk of bias in included studies

**Figure 2:**
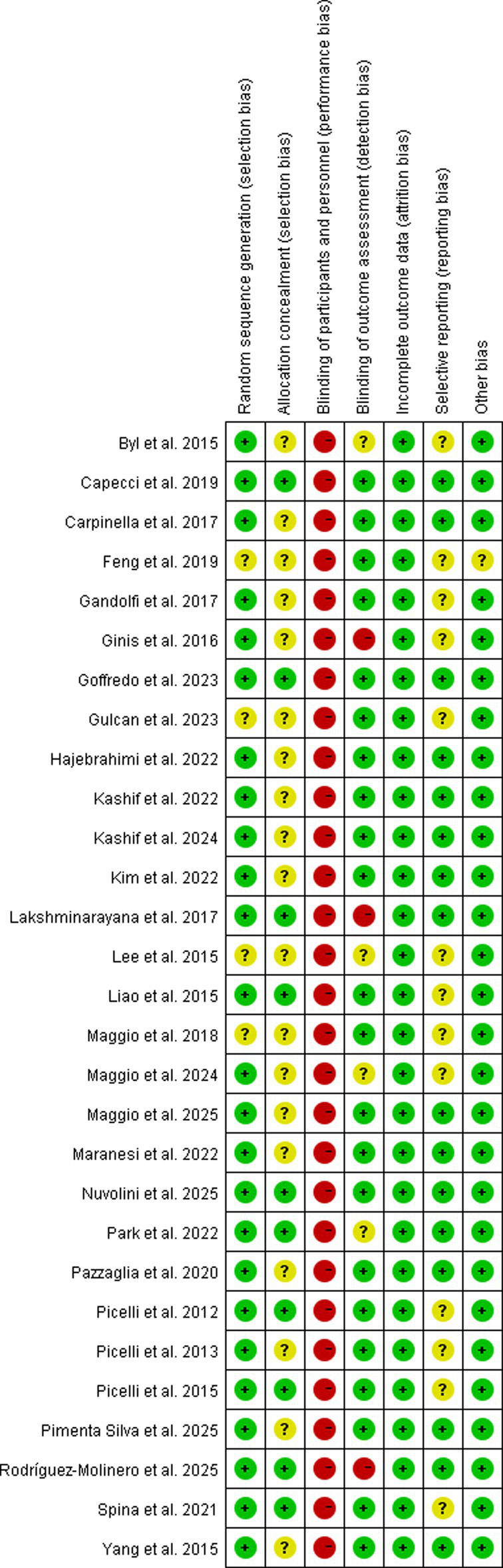
Risk of bias summary. Green circles indicate low risk of bias; yellow circles indicate unclear risk of bias; red circles indicate high risk of bias.

The overall risk of bias is presented in Figure 2. In this figure, green represents low risk of bias, yellow denotes unclear risk of bias, and red indicates high risk of bias. Specifically, 4 studies (13.8%) did not clearly report random sequence generation. Only 10 studies (34.5%) provided clear information on allocation concealment, and 19 studies (65.5%) were rated as having an unclear risk. All included studies were considered to carry a high risk of performance bias. With regard to detection bias, 3 studies (10.3%) were classified as high risk, 4 studies (13.8%) as unclear risk, and 22 studies (75.9%) as low risk. For attrition bias, 13 studies (44.8%) showed an unclear risk, whereas 16 studies (55.2%) were rated as low risk. As for reporting bias, 1 study (3.4%) was evaluated as unclear risk, and 28 studies (96.6%) as low risk.

### 3.4 Meta-analysis

#### 3.4.1 Balance function

**Figure 3:**
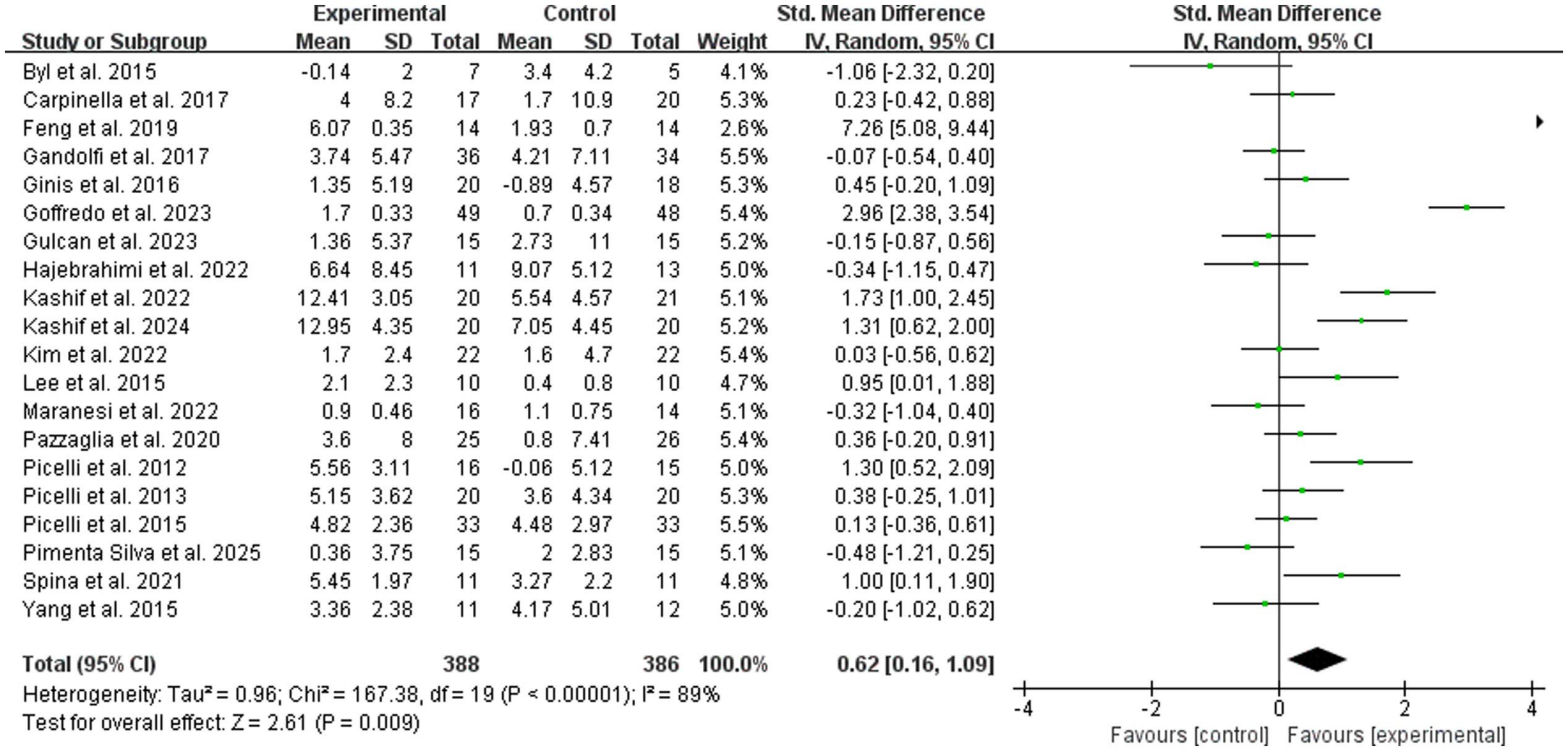
Forest plot of the effects of digital technology-based interventions on balance function in PD patients. SD, standard deviation; Std, standardized; IV, inverse variance; CI, confidence interval.

A total of 20 studies (9, 10, 13, 14, 17, 18, 27–34, 37–42) investigated the influence of digital technology-based interventions on balance function among patients with PD, and employed different assessment scales. The pooled effect size was estimated using the SMD across different assessment approaches. Meta-analysis results showed that digital technology-based interventions were associated with improved balance function in patients with PD versus the control group (*n* = 774, SMD = 0.62, 95% CI = 0.16 to 1.09, *p* = 0.009, I^2^ = 89%, Figure 3). However, substantial heterogeneity was observed, and the findings should be interpreted with caution.

#### 3.4.2 Cognitive function

**Figure 4:**
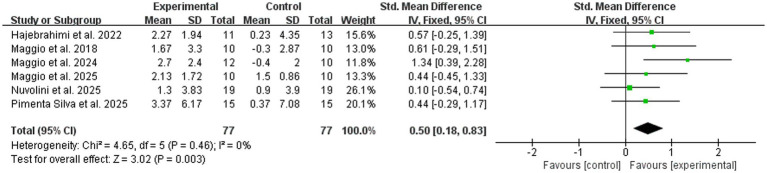
Forest plot of the effects of digital technology-based interventions on cognitive function in PD patients. SD, standard deviation; Std, standardized; IV, inverse variance; CI, confidence interval.

A total of 6 studies (14–16, 19, 36, 41) used different assessment scales to investigate the effect of digital technology-based interventions on cognitive function in patients with PD. Given the heterogeneity across assessment tools, SMD was adopted to calculate the pooled effect size. Meta-analysis revealed that patients with PD who received digital technology-based interventions showed better cognitive function than the control group (n = 154, SMD = 0.50, 95% CI 0.18 to 0.83, *p* = 0.003, I^2^ = 0%, Figure 4). However, the high risk of performance bias and unclear allocation concealment in the included studies limit the certainty of this finding.

#### 3.4.3 Depression

**Figure 5:**
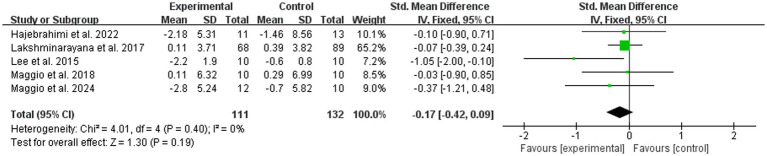
Forest plot of the effects of digital technology-based interventions on depression in PD patients. SD, standard deviation; Std, standardized; IV, inverse variance; CI, confidence interval.

A total of 5 studies (12, 14, 15, 17, 36) used different assessment scales to evaluate the effect of digital technology-based interventions on depression among individuals with PD. Given the variability in assessment tools, SMD was applied to quantify the pooled effect size. Meta-analysis results showed that, compared with the control group, digital technology-based interventions yielded no statistically significant improvements in depressive symptoms among patients with PD (*n* = 243, SMD = −0.17, 95% CI −0.42 to 0.09, *p* = 0.19, I^2^ = 0%, Figure 5).

#### 3.4.4 Quality of life

**Figure 6:**
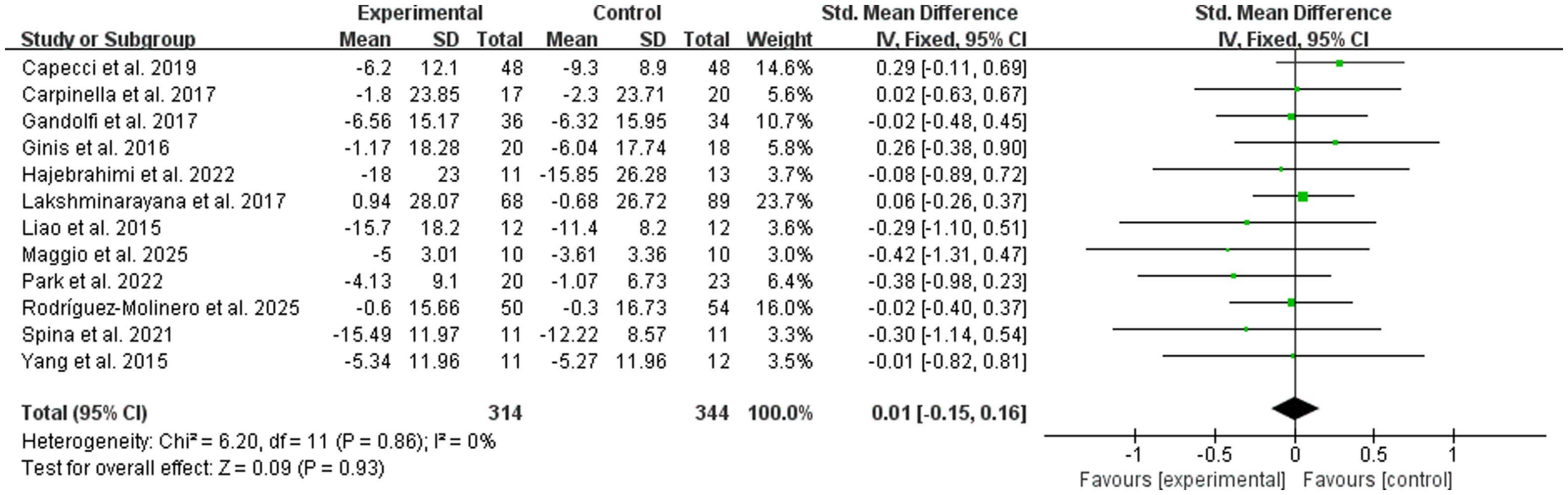
Forest plot of the effects of digital technology-based interventions on quality of life in PD patients. SD, standard deviation; Std, standardized; IV, inverse variance; CI, confidence interval.

The 12 studies included in this meta-analysis (10–12, 14, 16, 20, 26, 28, 30, 31, 35, 42) adopted different validated assessment scales. All these tools were used to assess the effect of digital technology-based interventions on the quality of life among individuals with PD. Given the differences in assessment instruments across studies, SMD was applied to quantify the pooled effect size. Meta-analysis results showed that, compared with the control group, digital technology-based interventions did not significantly improve the quality of life among individuals with PD (n = 658, SMD = 0.01, 95% CI − 0.15 to 0.16, *p* = 0.93, I^2^ = 0%, Figure 6).

### 3.5 Subgroup analyses

Our meta-analysis findings for balance function demonstrated substantial heterogeneity. Given the heterogeneity among the 20 included studies regarding intervention types, duration, frequency and intervention functional characteristics, we performed stratified subgroup analyses by these characteristics to explore the potential sources of heterogeneity. Furthermore, additional subgroup analyses for quality of life outcomes were undertaken according to intervention types.

#### 3.5.1 Subgroup analyses for balance function

We performed subgroup analyses of balance function based on four factors: intervention type, intervention duration, intervention frequency and intervention functional characteristics.

##### 3.5.1.1 Different intervention types

**Figure 7:**
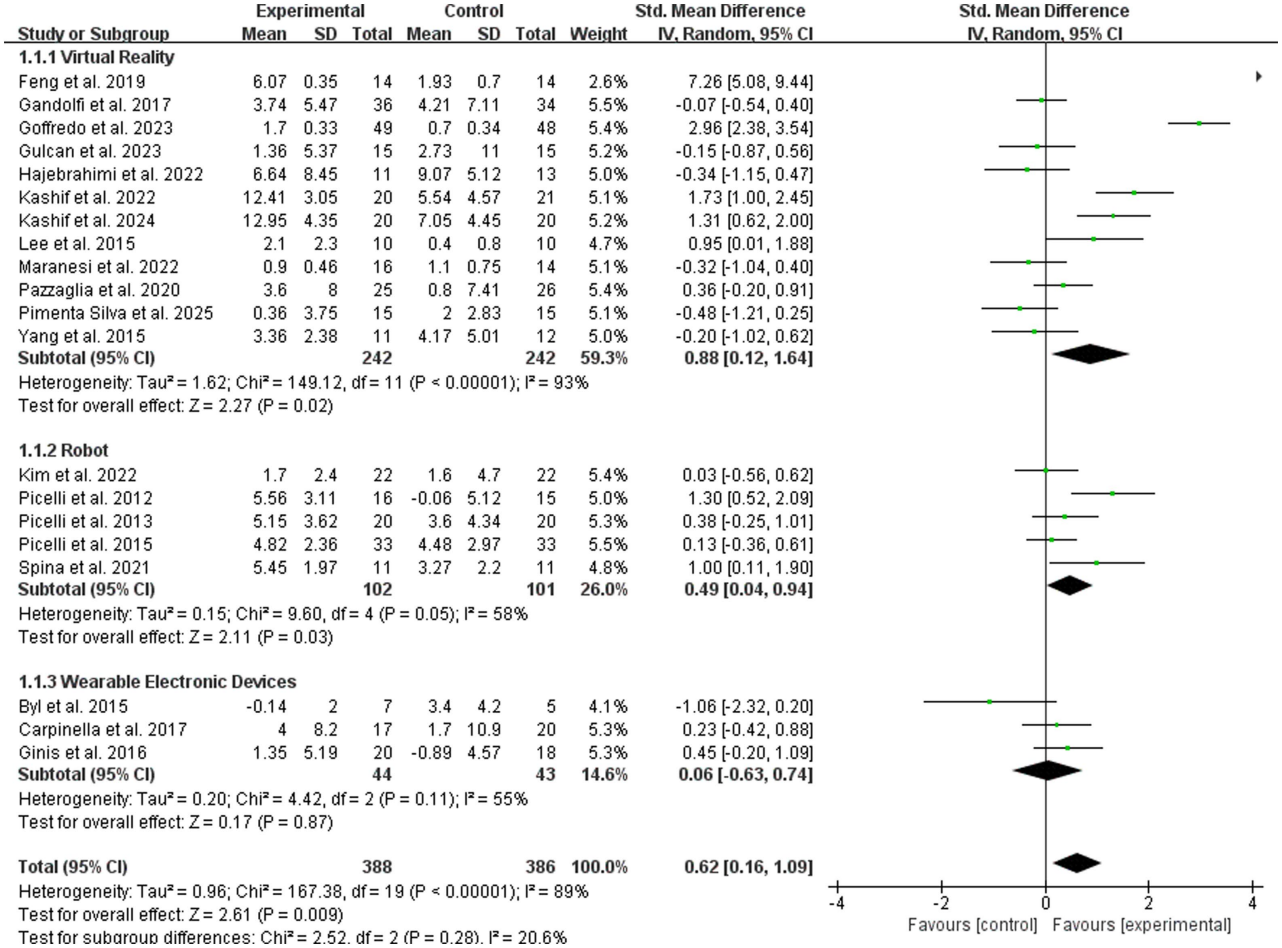
Forest plot of subgroup analysis of balance function by intervention type. SD, standard deviation; Std, standardized; IV, inverse variance; CI, confidence interval.

Subgroup analysis by intervention type (Figure 7) demonstrated that studies using virtual reality interventions were associated with improved balance function among patients with PD compared with the control group (*n* = 484, SMD = 0.88, *p* = 0.02). Studies employing robot-assisted training in the intervention group were also associated with improved balance function for patients with PD relative to the control group (*n* = 203, SMD = 0.49, *p* = 0.03). However, considerable heterogeneity was observed within both of these subgroups (I^2^ = 93% for virtual reality; I^2^ = 58% for robot-assisted training), and the findings should be interpreted with caution. By contrast, studies employing wearable devices in the intervention arm demonstrated no significant benefit in balance function among PD patients (*n* = 87, SMD = 0.06, *p* = 0.87).

##### 3.5.1.2 Different intervention duration

**Figure 8:**
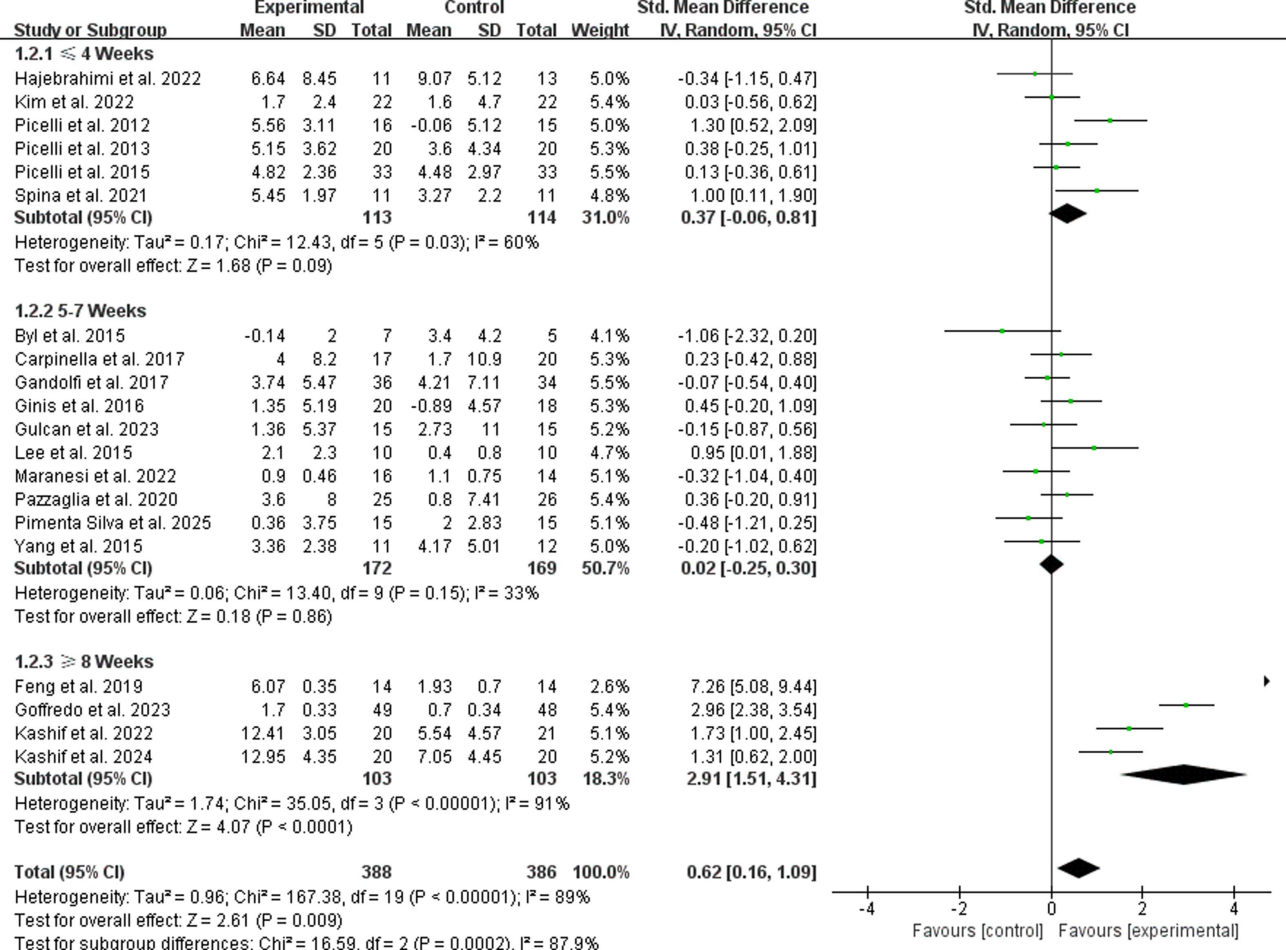
Forest plot of subgroup analysis of balance function by intervention duration. SD, standard deviation; Std, standardized; IV, inverse variance; CI, confidence interval.

Subgroup analysis stratified by intervention duration (Figure 8) revealed that intervention duration was a major source of heterogeneity in the meta-analysis of balance function. Digital technology-based interventions lasting ≥ 8 weeks were associated with enhanced balance function in PD patients (n = 206, SMD = 2.91, *p* < 0.0001). However, marked heterogeneity was present in this subgroup (I^2^ = 91%), requiring careful interpretation of the pooled result. By contrast, interventions with a duration of ≤ 4 weeks (*n* = 227, SMD = 0.37, *p* = 0.09) and 5–7 weeks (*n* = 341, SMD = 0.02, *p =* 0.86) did not yield statistically significant effects on balance function.

##### 3.5.1.3 Different intervention frequency

**Figure 9:**
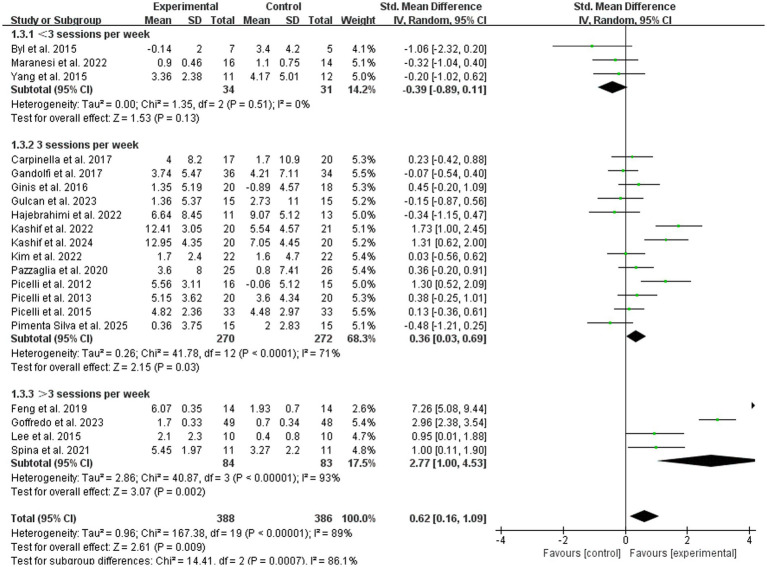
Forest plot of subgroup analysis of balance function by intervention frequency. SD, standard deviation; Std, standardized; IV, inverse variance; CI, confidence interval.

Subgroup analysis stratified by intervention frequency (Figure 9) demonstrated that intervention frequency was a major source of heterogeneity in the meta-analysis of balance function. Digital technology-based interventions delivered 3 times per week (*n* = 542, SMD = 0.36, *p* = 0.03) and > 3 times per week (*n* = 167, SMD = 2.77, *p* = 0.002) were both associated with improved balance function in PD patients. However, the >3 times weekly subgroup in particular showed an exceptionally large effect size and was based on a small number of studies, so the robustness of this specific finding requires further validation. In contrast, interventions administered < 3 times per week (*n* = 65, SMD = −0.39, *p = 0.13*) yielded no statistically significant improvement in balance function.

##### 3.5.1.4 Different intervention functional characteristics

**Figure 10:**
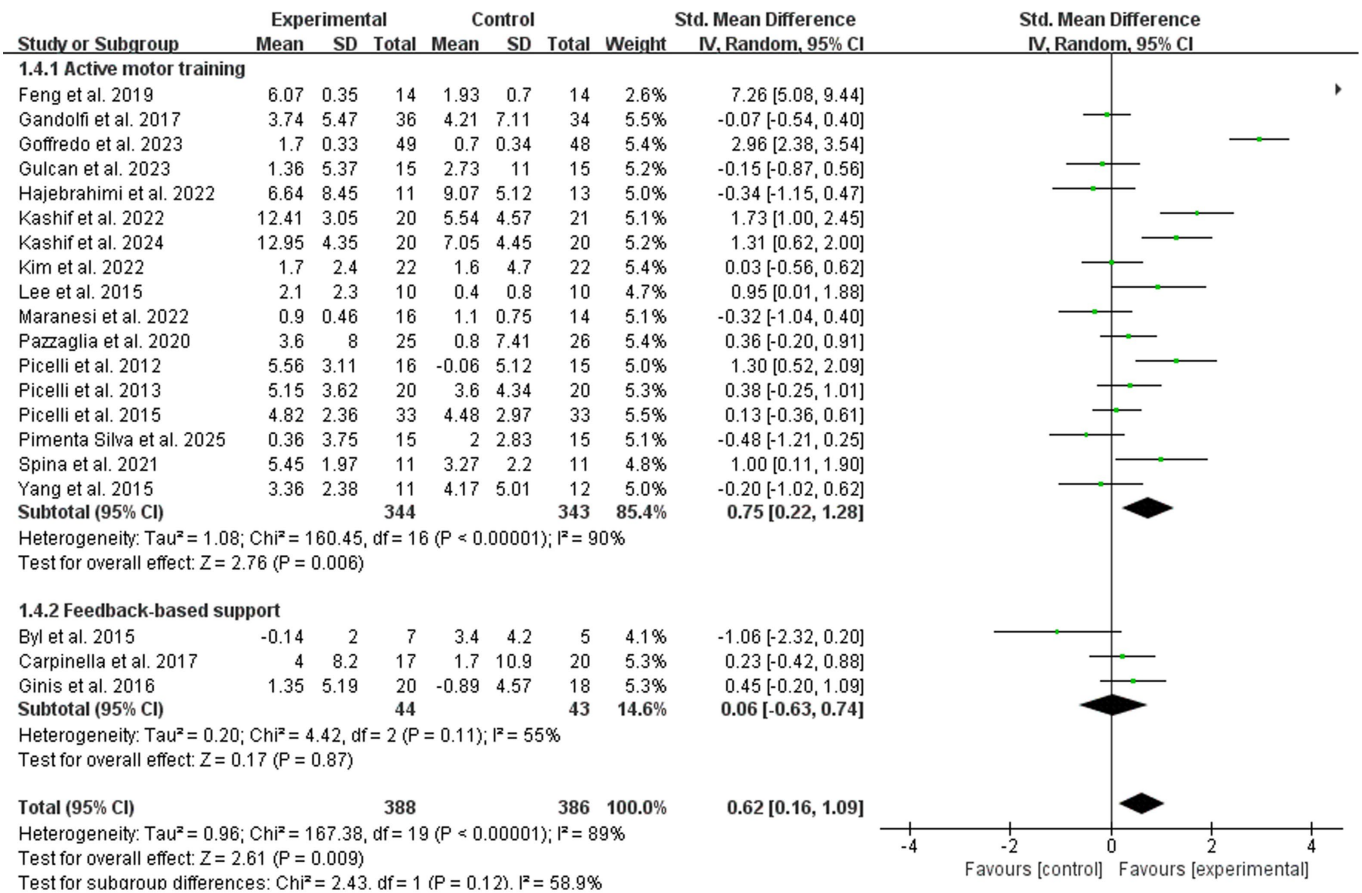
Forest plot of subgroup analysis of balance function by intervention functional characteristics. SD, standard deviation; Std, standardized; IV, inverse variance; CI, confidence interval.

According to the functional characteristics of interventions, the included studies were classified into two subgroups: active motor training and feedback support. Subgroup analysis was subsequently performed (Figure 10). The results showed that digital technology-based active motor training was associated with improved balance function in patients with Parkinson’s disease (*n* = 687, SMD = 0.75, *p* = 0.006). High heterogeneity was also observed in the active motor training subgroup (I^2^ = 90%), indicating that the differences in effect sizes across studies within this subgroup should not be overlooked. By contrast, digital technology-based feedback support did not show a statistically significant improvement in balance function (*n* = 87, SMD = 0.06, *p* = 0.87).

#### 3.5.2 Subgroup analyses for quality of life

**Figure 11:**
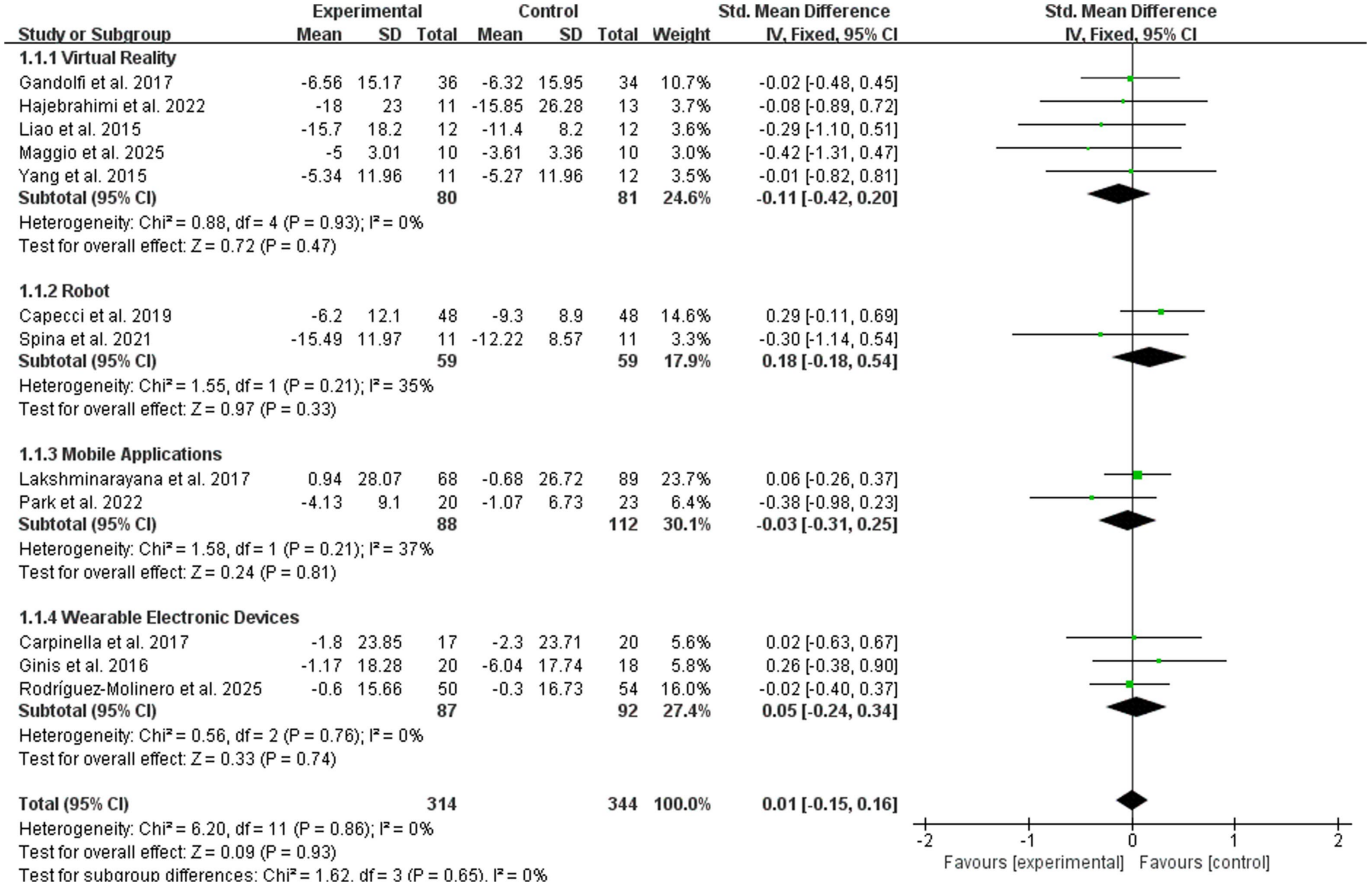
Forest plot of subgroup analysis of quality of life by intervention type. SD, standard deviation; Std, standardized; IV, inverse variance; CI, confidence interval.

Subgroup analysis stratified by intervention type (Figure 11) showed that virtual reality, robotic, mobile application and wearable electronic device interventions did not yield a statistically meaningful improvement in quality of life relative to the control group (*p* > 0.05).

### 3.6 Publication bias and sensitivity analysis

Visual assessment of funnel plots for meta-analyses with 10 or more included studies revealed no indication of publication bias (Supplementary Figure S1). We performed leave-one-out sensitivity analysis to assess the robustness of the pooled outcomes. For balance function, the outcome was unstable, with the statistical significance of the overall effect altered after exclusion of certain individual studies. In contrast, results for cognitive function, depression, and quality of life were robust, with no substantial changes observed following omission of any single study (Supplementary Figure S2).

## 4 Discussion

The present study is the first to comprehensively integrate data from global literature and summarize all available evidence on the efficacy of digital technology-based interventions for patients with PD. A total of 29 RCTs investigating digital technology-based interventions for PD were included in this study, and all studies employed an active control design. Active control is a routine choice in this field, which aligns with the clinical characteristics of PD and complies with ethical requirements for clinical research, enabling a direct comparison between digital technology-based interventions and conventional clinical interventions. This study provides a comprehensive and updated evidence base for the clinical practice and future research of digital technology-based interventions in PD.

In contrast to previous studies (21, 22), this study examined the effectiveness of various digital technology-based interventions for patients with PD. Meta-analysis demonstrated that digital technology-based interventions have the potential to improve balance function and cognitive function in patients with Parkinson’s disease. However, all included studies were at high risk of performance bias, and most studies had unclear allocation concealment, which severely limits the certainty of the pooled findings. High heterogeneity and unstable sensitivity analyses further indicate that the results are not robust and cannot be regarded as confirmatory. Meanwhile, these interventions exerted no statistically significant effects on depressive symptoms or quality of life.

This systematic review suggests that digital technology-based interventions have the potential to improve balance function in PD patients, while revealing substantial heterogeneity across different intervention strategies. Exploratory findings suggest that virtual reality technology and robot-assisted training might be associated with positive intervention effects. Existing evidence (43) indicates that virtual reality exerts its therapeutic effects via neurophysiological mechanisms encompassing multisensory integration, visual information processing mechanisms, and cortical activation, thereby bolstering postural control capacity and balance function in PD patients. Robot-assisted gait training (RAGT) adheres to the principles of intensive, high-dose, and task-specific training. It allows individualized and progressive adjustment of training difficulty according to patients’ actual performance, and provides both implicit and explicit feedback (44), making it suitable for PD patients with balance dysfunction. In recent years, wearable devices have been increasingly applied in the management of Parkinson’s disease to assess motor symptoms, monitor disease fluctuations, and assist with therapeutic interventions (45). Although the present systematic review did not identify a definitive beneficial effect of wearable devices on balance function, this technology holds great promise for Parkinson’s disease rehabilitation. It enables early screening of high-risk individuals and diagnosed patients, and demonstrates higher sensitivity than conventional assessment tools in monitoring disease progression (46).

Exploratory subgroup analyses suggested that digital technology-based interventions were associated with improvements in balance function when delivered for ≥8 weeks, at a frequency of ≥3 sessions per week, or as active exercise training. This pattern aligns with the observations of Lamichhane et al. (47) and He et al. (48), and points to a possible relationship between intervention dose and balance outcomes. At the same time, the sensitivity analysis was not robust, and heterogeneity across studies remained high.

The larger effect sizes seen in some subgroups appeared to come mainly from studies with small samples and methodological limitations. Including these studies increased the pooled estimates, contributed to heterogeneity, and made the sensitivity analysis unstable. So rather than reflecting true clinical benefit, these large estimates may simply reflect statistical uncertainty. Overall, these findings are best viewed as hypothesis-generating rather than practice-changing.

Given the small number of studies, considerable within-subgroup heterogeneity, unstable sensitivity results, and methodological concerns across trials, the true effect of digital technology-based interventions on balance function in Parkinson’s disease remains unclear. Larger, more rigorous studies are needed to clarify it.

For cognitive function, this systematic review demonstrated that digital technology-based interventions improved cognitive function in patients with PD. Such interventions exert effects through multidimensional cognitive stimulation and contextual interactive training, which can significantly suppress compensatory neural hyperactivity in brain regions including the prefrontal cortex. This mechanism optimizes the efficiency of cognitive resource allocation, promotes neuroplasticity, and thereby enhances overall cognitive performance (49). However, most existing studies have only employed virtual reality technology to improve cognitive function in this population (16, 19), and research involving other types of digital technologies remains scarce. Future large-scale randomized controlled trials covering a variety of digital technology modalities are therefore warranted to verify the effectiveness of digital interventions.

The present study found that digital technology-based interventions did not significantly alleviate depression in patients with PD. As a major non-motor symptom of PD, depression affects up to 38% of patients (50). However, most digital interventions focus primarily on improving motor symptoms and overlook the management of depressive symptoms (14, 17). To date, research on digital interventions for depression in this population remains limited, with insufficient evidence and substantial uncertainty in the findings.

Quality of life has become a key indicator for evaluating health status, guiding clinical decision-making, and assessing trial outcomes in PD patients (51). PD, a progressive neurodegenerative disorder, presents with a range of motor and non-motor symptoms, which significantly compromise patients’ physical functioning and reduce their overall quality of life. Although digital technology-based interventions hold promise for improving quality of life in PD patients, our findings indicate that their impact on quality of life was negligible. Furthermore, subgroup analyses showed that virtual reality, robotic training, mobile applications, and wearable electronic devices all failed to effectively improve quality of life in this population. This key indicator of daily well-being is influenced by multiple factors including motor function, mood, cognition, and social support. Improvement in a single domain can hardly lead to a significant enhancement in overall life quality. Furthermore, meaningful improvements in such outcomes require longer intervention and follow-up periods, whereas most existing digital interventions are relatively short in duration, making sustained benefits difficult to achieve. Further investigations are required to explore the influences of digital technology-based interventions on the life quality of PD patients.

The virtual reality training, robot-assisted rehabilitation, wearable electronic devices and mobile application interventions included in this review are inherently distinct clinical approaches. They differ markedly in core therapeutic mechanisms such as active motor training and feedback training, the extent of therapist involvement, and targeted clinical goals, and thus cannot be used interchangeably in clinical practice. Pooling these highly diverse interventions for meta-analysis inevitably introduces inherent clinical heterogeneity across all pooled outcomes, which serves as a major limitation of the present study.

Current evidence is methodologically limited and cannot be considered confirmatory, due to serious bias risks and high heterogeneity. Achieving double blinding of both participants and intervention providers is inherently challenging in rehabilitation interventions, so most included trials employed a single-blind design. This constraint confers a high risk of performance bias across all 29 included studies. Since most outcome measures relied on questionnaire-based assessments, this bias may inflate the estimated treatment effects. Meanwhile, poorly reported allocation concealment in most trials elevates the risk of selection bias, further eroding the overall certainty of the evidence.

Further investigation into the sources of heterogeneity showed that, beyond the previously described differences in intervention modalities, variations across studies in intervention duration, training frequency, and outcome measurement instruments also contributed to the observed high heterogeneity. Although we performed subgroup analyses stratified by intervention type, duration, frequency, and functional characteristics, considerable statistical heterogeneity persisted within subgroups. This finding suggests that in addition to discrepancies in intervention protocols, inconsistencies in patient characteristics, including disease stage, balance performance, and concomitant medication use, may collectively drive the residual heterogeneity.

Collectively, digital technology-based interventions hold promising value for improving balance function and non-motor outcomes in PD patients, yet their overall efficacy still requires validation through large-scale, high-quality randomized controlled trials.

## 5 Study limitations

This systematic review has certain inherent limitations. First, considerable methodological heterogeneity existed across the included studies, with substantial variability in experimental design, intervention strategies and outcome measurements. Although we attempted to control for heterogeneity via subgroup analyses and random-effects models, these statistical methods cannot fully eliminate its confounding effect on the pooled results. Second, it should be noted that potential confounding factors and disease-related characteristics, such as patient age, disease stage and comorbidities, may have influenced the findings, and these factors were not fully explored in our study. Third, all included studies were judged at high risk of performance bias, and allocation concealment was poorly reported in most trials, resulting in only moderate overall methodological quality. This limitation is particularly pronounced for subjective outcome measures and may lead to overestimation of the true treatment effect. In addition, subgroup analyses were not conducted for cognitive function and depression as secondary outcomes owing to the limited number of eligible studies.

## 6 Conclusion

Digital technology-based interventions may offer benefits for balance and cognitive function in patients with PD, though their effects on depression and quality of life remain unclear. For balance specifically, exploratory subgroup analyses pointed to potential improvements with virtual reality, robotic training, intervention duration of at least 8 weeks, a frequency of 3 or more sessions per week, and active exercise training. These findings, however, should be viewed as hypothesis-generating and require confirmation in future studies. The current evidence is limited by high heterogeneity, unstable sensitivity analysis for the primary outcome, high risk of performance bias across all included trials, and unclear allocation concealment in many studies. The evidence should therefore be regarded as preliminary and non-confirmatory. Whether digital technology-based interventions provide meaningful clinical benefit for PD rehabilitation will require larger, more rigorous trials.

## Data Availability

The original contributions presented in the study are included in the article/Supplementary material, further inquiries can be directed to the corresponding author.

## References

[1] Willis AW Roberts E Beck JC Fiske B Ross W Savica R . Incidence of Parkinson disease in North America. NPJ Parkinsons Dis. (2022) 8:170. doi: 10.1038/s41531-022-00410-y, 36522332 PMC9755252

[2] Tanner CM Ostrem JL. Parkinson's disease. N Engl J Med. (2024) 391:442–52. doi: 10.1056/NEJMra2401857, 39083773

[3] Armstrong MJ Okun MS. Diagnosis and treatment of Parkinson disease: a review. JAMA. (2020) 323:548–60. doi: 10.1001/jama.2019.22360, 32044947

[4] Mirelman A Bonato P Camicioli R Ellis TD Giladi N Hamilton JL . Gait impairments in Parkinson's disease. Lancet Neurol. (2019) 18:697–708. doi: 10.1016/S1474-4422(19)30044-4, 30975519

[5] Cong S Xiang C Zhang S Zhang T Wang H Cong S. Prevalence and clinical aspects of depression in Parkinson's disease: a systematic review and meta-analysis of 129 studies. Neurosci Biobehav Rev. (2022) 141:104749. doi: 10.1016/j.neubiorev.2022.104749, 35750224

[6] Zhang L Jia G Ma J Wang S Cheng L. Short and long-term effects of robot-assisted therapy on upper limb motor function and activity of daily living in patients post-stroke: a meta-analysis of randomized controlled trials. J Neuroeng Rehabil. (2022) 19:76. doi: 10.1186/s12984-022-01058-8, 35864524 PMC9306153

[7] Pearson C Hartzman A Munevar D Feeney M Dolhun R Todaro V . Care access and utilization among medicare beneficiaries living with Parkinson's disease. NPJ Parkinsons Dis. (2023) 9:108. doi: 10.1038/s41531-023-00523-y, 37429849 PMC10333279

[8] WHO Global Strategy on digital Health 2020–2025 World Health Organization (2021) Available online at: https://tinyurl.com/58d488cu (Accessed March 2026)

[9] Kashif M Albalwi AA Zulfiqar A Bashir K Alharbi AA Zaidi S. Effects of virtual reality versus motor imagery versus routine physical therapy in patients with parkinson's disease: a randomized controlled trial. BMC Geriatr. (2024) 24:229. doi: 10.1186/s12877-024-04845-1, 38443801 PMC10916168

[10] Spina S Facciorusso S Cinone N Armiento R Picelli A Avvantaggiato C . Effectiveness of robotic balance training on postural instability in patients with mild Parkinson's disease: a pilot, single blind, randomized controlled trial. J Rehabil Med. (2021) 53:jrm00154. doi: 10.2340/16501977-2793, 33585943 PMC8814837

[11] Rodríguez-Molinero A Pérez-López C Caballol N Buongiorno M Ávila Rivera MA López Ariztegui N . Parkinson's disease medication adjustments based on wearable device information compared to other methods: randomized clinical trial. NPJ Parkinsons Dis. (2025) 11:249. doi: 10.1038/s41531-025-00977-2, 40835832 PMC12368221

[12] Lakshminarayana R Wang D Burn D Chaudhuri KR Galtrey C Guzman NV . Using a smartphone-based self-management platform to support medication adherence and clinical consultation in Parkinson's disease. NPJ Parkinsons Dis. (2017) 3:2. doi: 10.1038/s41531-016-0003-z, 28649602 PMC5460235

[13] Goffredo M Baglio F DE Icco R Proietti S Maggioni G Turolla A . Efficacy of non-immersive virtual reality-based telerehabilitation on postural stability in Parkinson's disease: a multicenter randomized controlled trial. Eur J Phys Rehabil Med. (2023) 59:689–96. doi: 10.23736/S1973-9087.23.07954-6, 37847247 PMC10795069

[14] Hajebrahimi F Velioglu HA Bayraktaroglu Z Helvaci Yilmaz N Hanoglu L. Clinical evaluation and resting state fMRI analysis of virtual reality based training in Parkinson's disease through a randomized controlled trial. Sci Rep. (2022) 12:8024. doi: 10.1038/s41598-022-12061-3, 35577874 PMC9110743

[15] Maggio MG Luca A Cicero CE Calabrò RS Drago F Zappia M . Effectiveness of telerehabilitation plus virtual reality (tele-RV) in cognitive e social functioning: a randomized clinical study on Parkinson's disease. Parkinsonism Relat Disord. (2024) 119:105970. doi: 10.1016/j.parkreldis.2023.105970, 38142630

[16] Maggio MG Rizzo A Benenati A Mauro FG Cannavò A Pasquale PD. Efficacy and feasibility of virtual reality-based cognitive tele-rehabilitation in Parkinson's disease: a pilot randomized controlled trial on patients and caregivers. Digit Health. (2025) 11:20552076251376534. doi: 10.1177/20552076251376534 41018508 PMC12464430

[17] Lee NY Lee DK Song HS. Effect of virtual reality dance exercise on the balance, activities of daily living, and depressive disorder status of Parkinson's disease patients. J Phys Ther Sci. (2015) 27:145–7. doi: 10.1589/jpts.27.145, 25642060 PMC4305547

[18] Picelli A Melotti C Origano F Neri R Verzè E Gandolfi M . Robot-assisted gait training is not superior to balance training for improving postural instability in patients with mild to moderate Parkinson's disease: a single-blind randomized controlled trial. Clin Rehabil. (2015) 29:339–47. doi: 10.1177/0269215514544041, 25082957

[19] Nuvolini RA Silva KGD Freitas TB Doná F Torriani-Pasin C Pompeu JE. Exergame-based program and conventional physiotherapy based on Core areas of the European guideline similarly improve gait and cognition in people with Parkinson's disease: randomized clinical trial. Games Health J. (2025) 14:358–68. doi: 10.1089/g4h.2024.0116, 40160128

[20] Park Y Kim SR So HY Jo S Lee SH Hwang . Effect of mobile health intervention for self-management on self-efficacy, motor and non-motor symptoms, self-management, and quality of life in people with Parkinson's disease: randomized controlled trial. Geriatr Nurs (2022);46:90–97. doi:10.1016/j.gerinurse.2022.05.003, 35643018

[21] Fernandes S Oliveira B Sacadura S Rakasi C Furtado I Figueiredo JP . The effectiveness of virtual reality in improving balance and gait in people with Parkinson's disease: a systematic review. Sensors. (2025) 25:4795. doi: 10.3390/s25154795, 40807960 PMC12349033

[22] Wu D Yang Z Liu S Guan S Liu X Luo J. Effect of robot-assisted rehabilitation of patients with Parkinson's disease: a meta-analysis. Clin Rehabil. (2025) 39:1156–69. doi: 10.1177/02692155251355089, 40629925

[23] Liu R Zhang S Xiao Y Cheng Y Shang H. The effects of digital health interventions on motor symptoms, nonmotor symptoms, and quality of life in patients with Parkinson disease: systematic review and Meta-analysis of randomized controlled trials. J Med Internet Res. (2026) 28:e79935. doi: 10.2196/79935, 41818739 PMC13147926

[24] Page MJ McKenzie JE Bossuyt PM Boutron I Hoffmann TC Mulrow CD . The PRISMA 2020 statement: an updated guideline for reporting systematic reviews. BMJ. (2020) 372:n71. doi: 10.1136/bmj.n71, 33782057 PMC8005924

[25] Higgins JP Altman DG Gøtzsche PC Jüni P Moher D Oxman AD . The Cochrane collaboration's tool for assessing risk of bias in randomised trials. BMJ. (2011) 343:d5928. doi: 10.1136/bmj.d5928 22008217 PMC3196245

[26] Capecci M Pournajaf S Galafate D Sale P le Pera D Goffredo M . Clinical effects of robot-assisted gait training and treadmill training for Parkinson's disease. A randomized controlled trial. Ann Phys Rehabil Med. (2019) 62:303–12. doi: 10.1016/j.rehab.2019.06.016, 31377382

[27] Byl N Zhang W Coo S Tomizuka M. Clinical impact of gait training enhanced with visual kinematic biofeedback: patients with Parkinson's disease and patients stable post stroke. Neuropsychologia. (2015) 79:332–43. doi: 10.1016/j.neuropsychologia.2015.04.020, 25912760

[28] Carpinella I Cattaneo D Bonora G Bowman T Martina L Montesano A . Wearable sensor-based biofeedback training for balance and gait in Parkinson disease: a pilot randomized controlled trial. Arch Phys Med Rehabil. (2017) 98:622–630.e3. doi: 10.1016/j.apmr.2016.11.003, 27965005

[29] Feng H Li C Liu J Wang L Ma J Li G . Virtual reality rehabilitation versus conventional physical therapy for improving balance and gait in Parkinson's disease patients: a randomized controlled trial. Med Sci Monit. (2019) 25:4186–92. doi: 10.12659/MSM.916455, 31165721 PMC6563647

[30] Gandolfi M Geroin C Dimitrova E Boldrini P Waldner A Bonadiman S . Virtual reality telerehabilitation for postural instability in Parkinson's disease: a multicenter, single-blind, randomized, controlled trial. Biomed Res Int. (2017) 2017:7962826. doi: 10.1155/2017/7962826 29333454 PMC5733154

[31] Ginis P Nieuwboer A Dorfman M Ferrari A Gazit E Canning CG . Feasibility and effects of home-based smartphone-delivered automated feedback training for gait in people with Parkinson's disease: a pilot randomized controlled trial. Parkinsonism Relat Disord. (2016) 22:28–34. doi: 10.1016/j.parkreldis.2015.11.004 26777408

[32] Gulcan K Guclu-Gunduz A Yasar E Ar U Sucullu Karadag Y Saygili F. The effects of augmented and virtual reality gait training on balance and gait in patients with Parkinson's disease. Acta Neurol Belg. (2023) 123:1917–25. doi: 10.1007/s13760-022-02147-0, 36443623 PMC9707084

[33] Kashif M Ahmad A Bandpei MAM Gilani SA Hanif A Iram H. Combined effects of virtual reality techniques and motor imagery on balance, motor function and activities of daily living in patients with Parkinson's disease: a randomized controlled trial. BMC Geriatr. (2022) 22:381. doi: 10.1186/s12877-022-03035-1, 35488213 PMC9055773

[34] Kim H Kim E Yun SJ Kang MG Shin HI Oh BM . Robot-assisted gait training with auditory and visual cues in Parkinson's disease: a randomized controlled trial. Ann Phys Rehabil Med. (2022) 65:101620. doi: 10.1016/j.rehab.2021.101620, 34896605

[35] Liao YY Yang YR Cheng SJ Wu YR Fuh JL Wang RY. Virtual reality-based training to improve obstacle-crossing performance and dynamic balance in patients with Parkinson's disease. Neurorehabil Neural Repair. (2015) 29:658–67. doi: 10.1177/1545968314562111, 25539782

[36] Maggio MG De Cola MC Latella D Maresca G Finocchiaro C La Rosa G . What about the role of virtual reality in Parkinson disease's cognitive rehabilitation? Preliminary findings from a randomized clinical trial. J Geriatr Psychiatry Neurol. (2018) 31:312–8. doi: 10.1177/0891988718807973 30360679

[37] Maranesi E Casoni E Baldoni R Barboni I Rinaldi N Tramontana B . The effect of non-immersive virtual reality exergames versus traditional physiotherapy in Parkinson's disease older patients: preliminary results from a randomized-controlled trial. Int J Environ Res Public Health. (2022) 19:14818. doi: 10.3390/ijerph192214818 36429537 PMC9690935

[38] Pazzaglia C Imbimbo I Tranchita E Minganti C Ricciardi D Lo Monaco R . Comparison of virtual reality rehabilitation and conventional rehabilitation in Parkinson's disease: a randomised controlled trial. Physiotherapy. (2020) 106:36–42. doi: 10.1016/j.physio.2019.12.007, 32026844

[39] Picelli A Melotti C Origano F Waldner A Gimigliano R Smania N. Does robotic gait training improve balance in Parkinson's disease? A randomized controlled trial. Parkinsonism Relat Disord. (2012) 18:990–3. doi: 10.1016/j.parkreldis.2012.05.010, 22673035

[40] Picelli A Melotti C Origano F Neri R Waldner A Smania N. Robot-assisted gait training versus equal intensity treadmill training in patients with mild to moderate Parkinson's disease: a randomized controlled trial. Parkinsonism Relat Disord. (2013) 19:605–10. doi: 10.1016/j.parkreldis.2013.02.010, 23490463

[41] Pimenta Silva D Bouça-Machado R Pona-Ferreira F Lobo T Cacho R Anker R . Combining immersive exergaming with physiotherapy in a specialized intensive Parkinson's disease rehabilitation program: a randomized controlled trial. J Neuroeng Rehabil. (2025) 22:131. doi: 10.1186/s12984-025-01640-w, 40500714 PMC12153140

[42] Yang WC Wang HK Wu RM Lo CS Lin KH. Home-based virtual reality balance training and conventional balance training in Parkinson's disease: a randomized controlled trial. J Formos Med Assoc. (2016) 115:734–43. doi: 10.1016/j.jfma.2015.07.012, 26279172

[43] Agostini F Conti M Morone G Iudicelli G Fisicaro F Savina A . The role of virtual reality in postural rehabilitation for patients with Parkinson's disease: a scoping review. Brain Sci. (2024) 15:23. doi: 10.3390/brainsci15010023 39851391 PMC11764033

[44] Hotz I Mildner S Stampfer-Kountchev M Slamik B Blättner C Türtscher E . Robot-assisted gait training in patients with various neurological diseases: a mixed methods feasibility study. PLoS One. (2024) 19:e0307434. doi: 10.1371/journal.pone.0307434 39190743 PMC11349200

[45] Hirczy S Zabetian C Lin YH. The current state of wearable device use in Parkinson's disease: a survey of individuals with Parkinson's. Front Digit Health. (2024) 6:1472691. doi: 10.3389/fdgth.2024.1472691 39764207 PMC11701158

[46] Adams JL Lizarraga KJ Waddell EM Myers TL Jensen-Roberts S Modica JS . Digital Technology in Movement Disorders: updates, applications, and challenges. Curr Neurol Neurosci Rep. (2021) 21:16. doi: 10.1007/s11910-021-01101-6, 33660110 PMC7928701

[47] Lamichhane P Sukralia S Alam B Shaikh S Farrukh S Ali S . Augmented reality-based training versus standard training in improvement of balance, mobility and fall risk: a systematic review and meta-analysis. Ann Med Surg. (2023) 85:4026–32. doi: 10.1097/MS9.0000000000000986, 37554880 PMC10406051

[48] He Z Wu H Zhao G Zhang Y Li C Xing Y . The effectiveness of digital technology-based Otago exercise program on balance ability, muscle strength and fall efficacy in the elderly: a systematic review and meta-analysis. BMC Public Health. (2025) 25:71. doi: 10.1186/s12889-024-21251-9, 39773392 PMC11707927

[49] Liao YY Tseng HY Lin YJ Wang CJ Hsu WC. Using virtual reality-based training to improve cognitive function, instrumental activities of daily living and neural efficiency in older adults with mild cognitive impairment. Eur J Phys Rehabil Med. (2020) 56:47–57. doi: 10.23736/S1973-9087.19.05899-4, 31615196

[50] Zhao J Jia H Ma P Zhu D Fang Y. Multidimensional mechanisms of anxiety and depression in Parkinson's disease: integrating neuroimaging, neurocircuits, and molecular pathways. Pharmacol Res. (2025) 215:107717. doi: 10.1016/j.phrs.2025.107717, 40157405

[51] Bock MA Brown EG Zhang L Tanner C. Association of Motor and Nonmotor Symptoms with Health-Related Quality of life in a large online cohort of people with Parkinson disease. Neurology. (2022) 98:e2194–203. doi: 10.1212/WNL.0000000000200113, 35418456 PMC9162165

