# Supplementary material for: Effects of digital technology-based interventions on balance function and non-motor outcomes in Parkinson’s disease: a systematic review and meta-analysis
Source: Front Neurol. 2026 Jul 29;17:1844772. doi: 10.3389/fneur.2026.1844772 (PMC13462945; doi:10.3389/fneur.2026.1844772)
Supplement: Supplementary file 1 [file Supplementary_file_1.docx]

**Figure S1. Assessment of potential publication bias by funnel plots**

(a) **Balance function (b)Quality of Life**

(a) Balance function

(b) Quality of Life

**Figure S2 Sensitivity analysis**

**(a)Balance function (b)Cognitive function (c)Depression (d) Quality of Life**

(a) Balance function

(b) Cognitive function

(c) Depression

(d) Quality of Life
