# Supplementary material for: Effects of digital technology-based interventions on balance function and non-motor outcomes in Parkinson’s disease: a systematic review and meta-analysis
Source: Front Neurol. 2026 Jul 29;17:1844772. doi: 10.3389/fneur.2026.1844772 (PMC13462945; doi:10.3389/fneur.2026.1844772)
Supplement: Supplementary file 2 [file Table_1.docx]

| **Table S1 Characteristics of included studies** | | | | | | | | | | |
| --- | --- | --- | --- | --- | --- | --- | --- | --- | --- | --- |
| **Study ID** | **Country** | **Age (years)** | **Number of participants** | | **Interventions** | | **Blinding** | **Control Type** | **Evaluation Time Points** | **Duration** |
|  |  |  | Ex | C | Ex | C |  |  |  |  |
| Byl et al.  2015 [27] | USA | Ex：68.5 (3.6)  C：70 (2.9) | 7 | 5 | Usual gait training plus kinematic feedback | Usual gait training | Not Reported | Active Control | Baseline、posttreatment | 6 weeks |
| Capecci et al.  2019 [26] | Italy | Ex：68.1 (9.8)  C：67.0 (7.6) | 48 | 48 | Robot-assisted gait training | Treadmill training | SB | Active Control | Baseline、posttreatment | 4 weeks |
| Carpinella et al. 2017 [28] | Italy | Ex：73.0 (7.1)  C：75.6 (8.2) | 17 | 20 | Wearable sensor-based feedback training | Individually structured physiotherapy without feedback | SB | Active Control | Preintervention,postintervention,and at 1-month follow-up | 6-7 weeks |
| Feng et al. 2019 [29] | China | Ex:67.47(4.79)  C:66.93(4.64) | 14 | 14 | Virtual Reality Rehabilitation | Conventional Physical Therapy | SB | Active Control | Baseline, Posttreatment | 12 weeks |
| Gandolfi et al.  2017 [30] | Italy | Ex：67.45 (7.18)  C：69.84 (9.41) | 36 | 34 | Remotely supervised in home VR balance training | In-clinic sensory integration balance training (SIBT) | SB | Active Control | Baseline, Postintervention, 1-Month Follow-up | 7 weeks |
| Ginis et al.  2016 [31] | Belgium | Ex：67.30 (8.13)  C：66.11 (8.07) | 20 | 18 | Home-based smartphone-delivered automated feedback training | Traditional gait training | U | Active control | Baseline, Postintervention, 4-Week Follow-up | 6 weeks |
| Goffredo et al.  2023 [13] | Italy | Ex：67.8(6.6)  C：68.2(5.8) | 49 | 48 | non-immersive  virtual reality-  based telerehabilitation | At-home self-administered structured conventional motor activities | SB | Active control | Baseline, Posttreatment | 6-10 weeks |
| Gulcan et al.  2023 [32] | Turkey | Ex：61.63 (7.36)  C：59.27 (13.09) | 15 | 15 | AR and VR gait training combined with conventional training | Conventional training | SB | Active Control | Baseline、Posttreatment | 6 weeks |
| Hajebrahimi et al.  2022 [14] | Turkey | Ex：66.36 (8.04)  C：65.53 (9.93) | 11 | 13 | Virtual Reality based Exergaming | Exercise Therapy | SB | Active Control | Baseline、Posttreatment | 4 weeks |
| Kashif et al.  2022 [33] | Pakistan | Ex：63.86(4.57)  C：62.32(4.61) | 20 | 21 | Virtual Reality techniques and motor imagery in addition to physical therapy | Routine physical therapy | SB | Active Control | Baseline, 6th Treatment Week, 12th Treatment Week, 16th Follow-up Week | 12 weeks |
| Kashif et al.  2024 [9] | Pakistan | Ex：63.20 (4.85)  C：61.95 (4.62) | 20 | 20 | Virtual Reality therapy in addition to routine physical therapy | Routine physical therapy | SB | Active Control | Baseline, 6th Therapy Week, 12th Therapy Week, 16th Follow-up Week | 12 weeks |
| Kim et al. 2022 [34] | Korea | Ex: 68.7 (6.9)  C: 67.5 (9.3) | 22 | 22 | Robot-Assisted Gait Training (RAGT) | Treadmill Training (TT) | SB | Active Control | Baseline, Posttreatment, 1 month after treatment | 4 weeks |
| Lakshminarayana et al.2017 [12] | UK | Ex：59.86 (9.13)  C：60.71 (10.26) | 68 | 89 | Smartphone-based self-management platform | Conventional therapy | U | Active Control | Baseline,  16 weeks | 16 weeks |
| Lee et al.  2015 [17] | Korea | Ex：68.4(2.9)  C：70.1(3.3) | 10 | 10 | Virtual Reality dance exercise plus conventional therapy | Conventional therapy | Not Reported | Active Control | Baseline,  Posttreatment | 6 weeks |
| Liao et al. 2015 [35] | China | Ex: 67.3 (7.1)  C: 65.1(6.7) | 12 | 12 | Virtual Reality–based Wii Fit exercise | Traditional exercise | SB | Active Control | Baseline,  Posttreatment,  30-day post-intervention (follow-up) | 6 weeks |
| Maggio et al.  2018 [36] | Italy | Ex：69.9 (6.3)  C：68.9 (10.05) | 10 | 10 | Semi-immersivevirtual reality training | Traditional cognitive training | SB | Active Control | Baseline, Posttreatment | 8 weeks |
| Maggio et al.  2024 [15] | Italy | Ex：66.8 (6.5)  C：63.8 (8.3) | 12 | 10 | Mobile Applications plus virtual reality | Traditional cognitive training | Not Reported | Active Control | Baseline,  Posttreatment,3-months follow-up | 6 weeks |
| Maggio et al.  2025 [16] | Italy | Ex：63.6 (2.6)  C：63.8 (2.0) | 10 | 10 | Tele-rehabilitation plus virtual reality | Traditional cognitive training | SB | Active Control | Baseline, Posttreatment | 8 weeks |
| Maranesi et al.  2022 [37] | Italy | Ex：72.7 (6.3)  C：75.5 (5.4) | 16 | 14 | Non-immersive  virtual reality  Therapy plus traditional rehabilitation | Traditional rehabilitation | SB | Active Control | Baseline, Posttreatment | 5 weeks |
| Nuvolini et al. 2025 [19] | Brazil | Ex: 62.7 (6.8)  C: 69.2 (7.8) | 19 | 19 | Training based on Kinect exergames | Conventional physiotherapeutic training | SB | Active control | Baseline, Posttreatment,30-day post-intervention (follow-up) | 7 weeks |
| Park et al.  2022 [20] | Korea | Ex：62.20(7.43)  C：64.27(8.28) | 20 | 23 | Mobile Applications plus Smartwatches | Short text messages and telephone counselling | Not Reported | Active control | Baseline, Posttreatment | 16 weeks |
| Pazzaglia et al.  2020 [38] | Italy | Ex：72 (7)  C：70 (10) | 25 | 26 | Virtual Reality rehabilitation | Conventional rehabilitation | SB | Active control | Baseline、Posttreatment | 6 weeks |
| Picelli et al.  2012 [39] | Italy | 68.3 | 16 | 15 | Robot-assisted gait training | Physical Therapy | SB | Active control | Baseline, Post-treatment, 1-Month Post-treatment | 4 weeks |
| Picelli et al. 2013 [40] | Italy | Ex:68.50 (10.10)  C:68.80 (7.72) | 20 | 20 | Robot-assisted gait training | Treadmill Training | SB | Active control | Baseline, Post-treatment,3 months post-treatment | 4 weeks |
| Picelli et al.  2015 [18] | Italy | Ex：68.2 (9.2)  C：69.7 (7.2) | 33 | 33 | Robotic gait training | Balance training | SB | Active control | Baseline, Posttreatment, 1-Month Posttreatment | 4 weeks |
| Pimenta Silva et al. 2025 [41] | Portugal | Ex: 63[54, 71]  C: 70[66, 76] | 15 | 15 | MindPod Dolphin (virtual reality intervention) plus physiotherapy | Physiotherapy | SB | Active control | Baseline,  Posttreatment | 6 weeks |
| Rodríguez-Molinero et al. 2025 [11] | Spain | Ex:65.9 (8.85)  62.5: (9.05) | 50 | 54 | Parkinson’s Holter (wearable sensor)+Routine care | Routine clinical care | TB | Active control | Baseline,  12 weeks post-intervention (±2 weeks),  26 weeks post-intervention (±2 weeks) | 26 weeks |
| Spina et al.  2021 [10] | Italy | Ex：68 (6.9)  C：67.27 (4.85) | 11 | 11 | Robotic balance training | conventional balance training | SB | Active control | Baseline, Postintervention, and at 1-month follow-up | 4 weeks |
| Yang et al.  2016 [42] | China | Ex：72.5 (8.4)  C：75.4 (6.3) | 11 | 12 | Home-based virtual reality balance training | Conventional balance training | SB | Active control | Baseline, Week 6 (posttest), and Week 8 (follow-up) | 6 weeks |
